# A global database for climate-related financial policies

**DOI:** 10.1186/s13104-023-06418-8

**Published:** 2023-07-06

**Authors:** Paola D’Orazio

**Affiliations:** Chair of Economics, Faculty of Economics and Business Administration, Technische Universität Chemnitz, Chemnitz, Germany

**Keywords:** Climate-related financial policies, Climate risks, Climate change, Financial stability, Prudential regulations, Green financial policymaking, Database

## Abstract

**Objectives:**

This article introduces the Climate-related Financial Policies Database and provides statistics on its main indicators. The database records many aspects of green financial policymaking for 74 nations for the period 2000–2020 by financial (central banks, financial regulators, supervisors) and non-financial (ministries, banking organizations, governments, and others) entities. The database is crucial for identifying and evaluating present and future trends in green financial policies, as well as the role played by central banks and regulators in raising green financing and taming financial instability caused by climate change.

**Data description:**

The database captures various aspects of financial (central banks and financial regulators and supervisors) and non-financial institutions' (ministries, banking associations, governments, and others) green financial policymaking in the period 2000–2020. Information is collected for the following variables: country/jurisdiction, economic development level (as defined by the World Bank Indicators), year of policy adoption, measure adopted and its bindingness, and authority/ies responsible for its implementation The database includes 74 countries, of which 39 are advanced economies, 20 are emerging, and 15 are developing economies. Open knowledge and data sharing encouraged by this article can support research in the developing field of financial policymaking related to climate change.

## Objective

Extreme weather events can result in financial losses for non-financial businesses and increase their financial fragility, coupled with substantial economic costs [1]. Due to financial and economic losses, the destruction of production capital, the fall in profitability of exposed enterprises, and the stranding of assets associated with climate-relevant industries, climate-related financial risks can result in credit, market, liquidity, and insurance risks [2]. Policies must therefore be designed to reduce the effects of financial sector fragility in the face of such threats; increasing our understanding of the policies used on a global scale would aid in this effort.

Our knowledge of what policies are used across countries and time, which the authorities responsible for their promotion and implementation, and the “bindingness” of the policy is, however, still quite limited, hindering our ability to understand the newly developed financial policies and institutions’ reforms in the short and medium run, in response to climate change threats to the financial system stability.

The database aims to close the significant data gap related to climate-related financial policies (CRFPs) for academics and decision-makers, which currently hampers the assessment of the impact of the reforms fostered by central banks and financial regulators on climate-related risks.

The database permits the construction of the CRFP index to assess, quantify, and compare international engagement to climate-related financial policymaking in recent decades [3] and the analysis of pandemic and climate change threats in the prudential regulatory frameworks of G20 countries [4]. Moreover, the different indicators of Policy Areas contained in the database have been used to investigate whether these policies contribute to the reduction of CO2 emission in G20 countries [5], to study the empirical link between the climate-related financial policies and the central bank and/or financial regulators governance structure [6] and which factors influence the decision to adopt climate-related financial policies [7].

### Data description

**Fig. 1:**
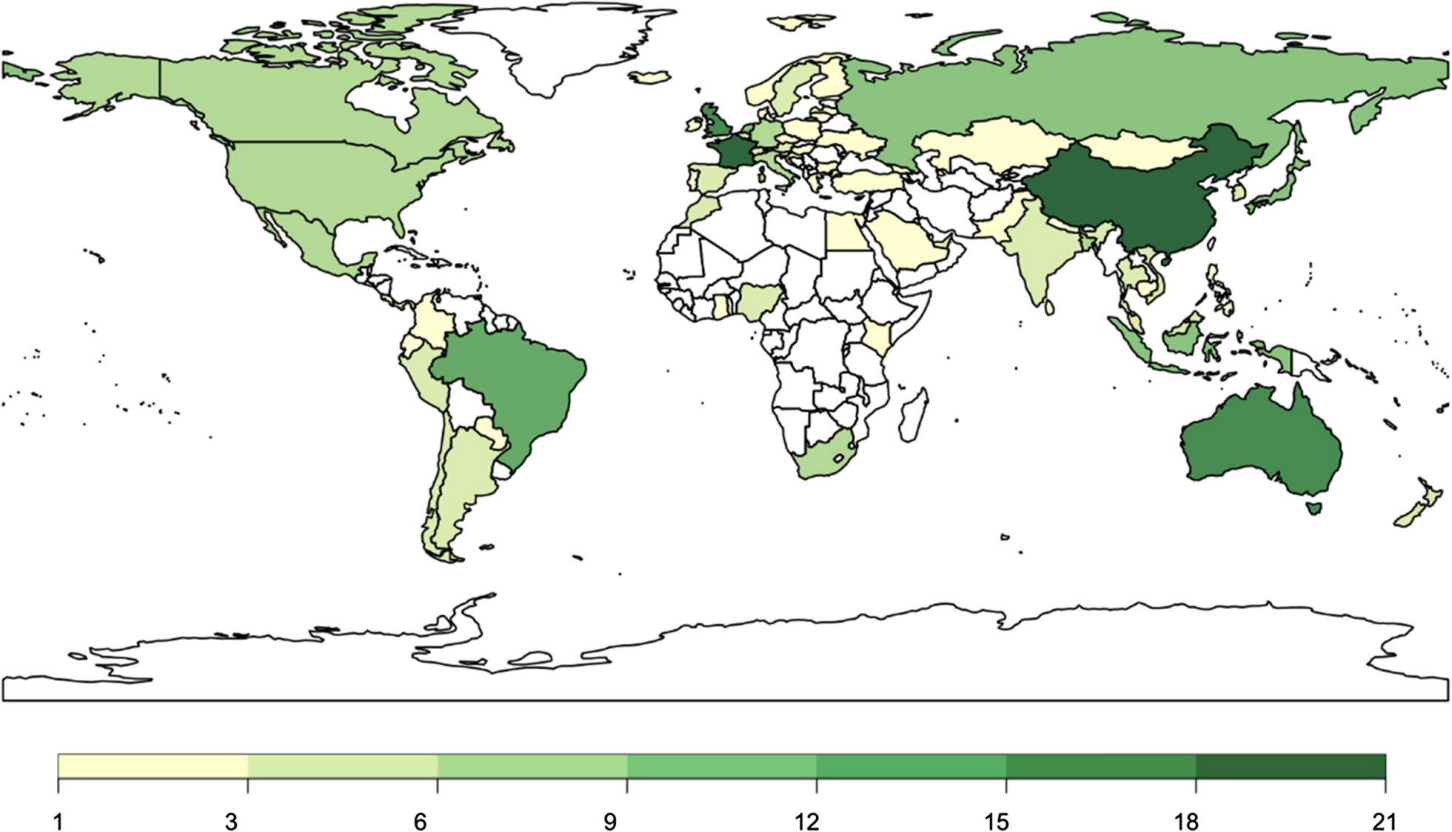
Spatial coverage of the database and the total number of policies adopted by each country as of December 2020. Source: author elaboration

**Fig. 2:**
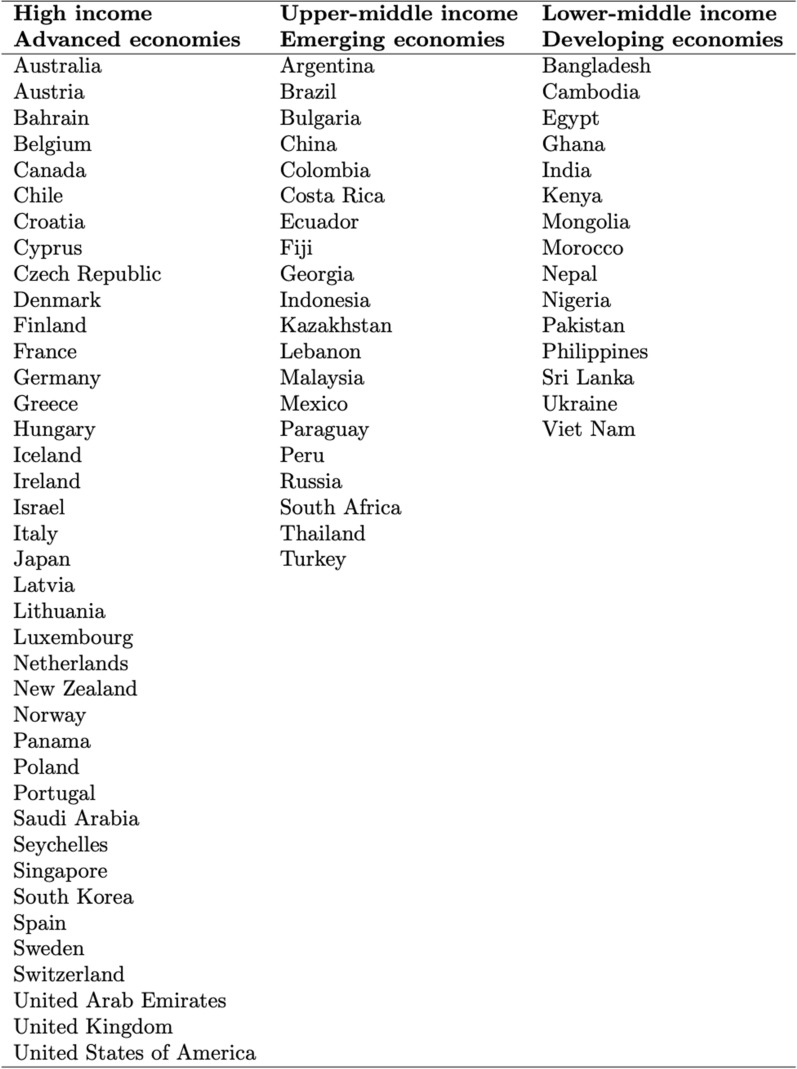
Countries classification by income group—World Bank classification

This research builds a collection of global CRFPs issued by multiple authorities from 2000 to 2020 by retrieving policy documents from official databases and websites. The database covers 74 countries, of which 39 are advanced, 20 are emerging, and 15 are developing economies (Fig. 1 [Data file 3] and Fig. 2 [Data file 4]).

**Fig. 3:**
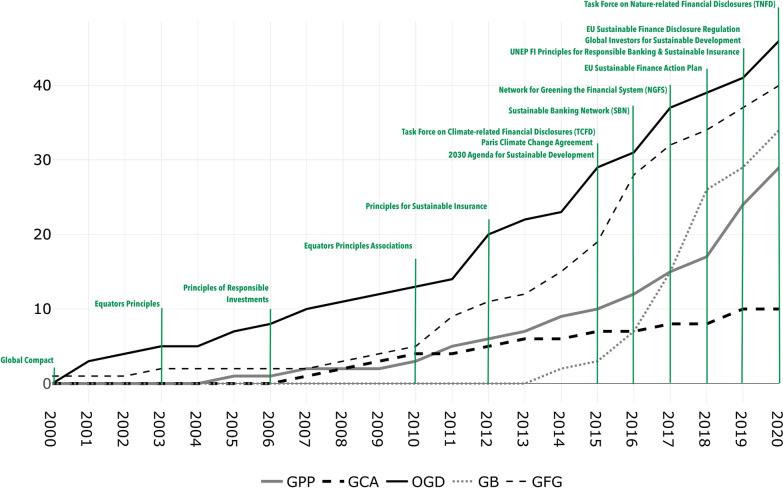
Cumulated adoption of climate-related financial policies. Source: author elaboration

Collected data shows that the number of policies adopted each year has risen over time, reflecting a greater level of participation among nations. Evidence collected in the database shows that globally the financial sector has been more involved in integrating standards and policy efforts and promoting financial industry transparency and disclosure standards following an increased international engagement (Fig. 3 [Data file 5]).

**Fig. 4:**
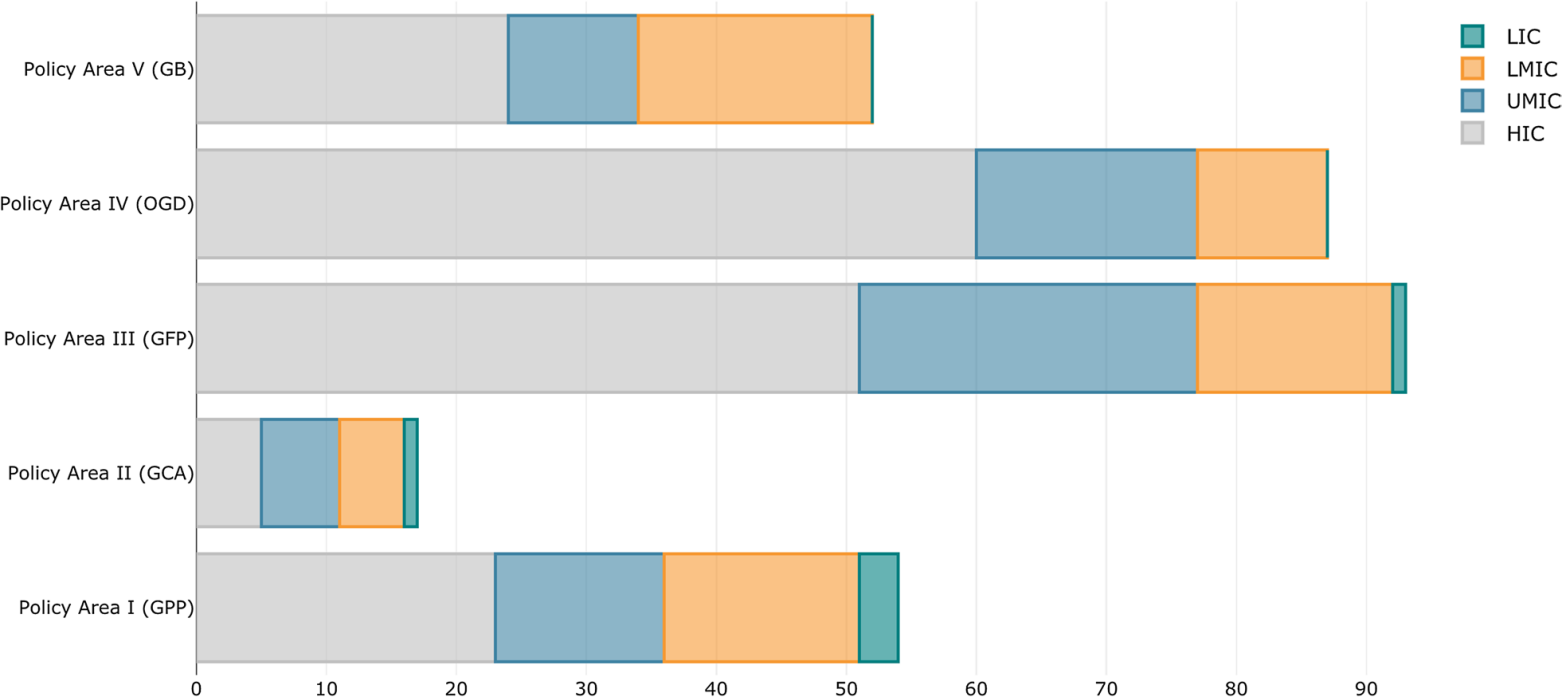
Measures—sorted by Policy Areas—adopted by different income groups. Source: author elaboration

Disclosure requirements for non-financial institutions and green financial policies are the most common and adopted by high-income countries. Upper-middle-income countries are characterized by high adoption of green-financial policies, followed by disclosure requirements and green prudential policies. Low-middle-income countries have been more active in promoting green bonds, followed by green prudential regulations and financial principles. Low-income countries account for the lowest share of overall adoption; a higher engagement in Policy Area I is instead reported for these countries (Fig. 4 [Data file 6]).

**Fig. 5:**
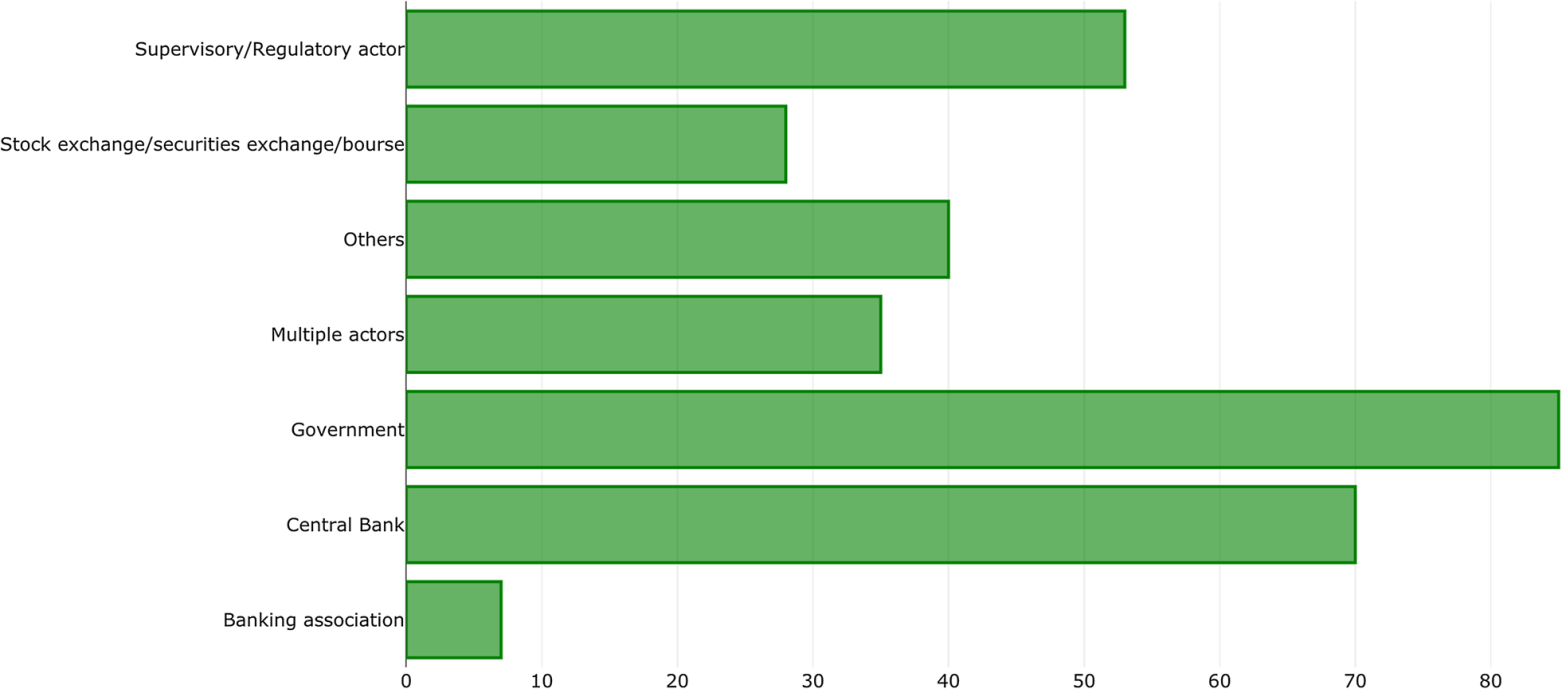
Distribution of policy adoption by bindingness. Source: author elaboration

**Fig. 6:**
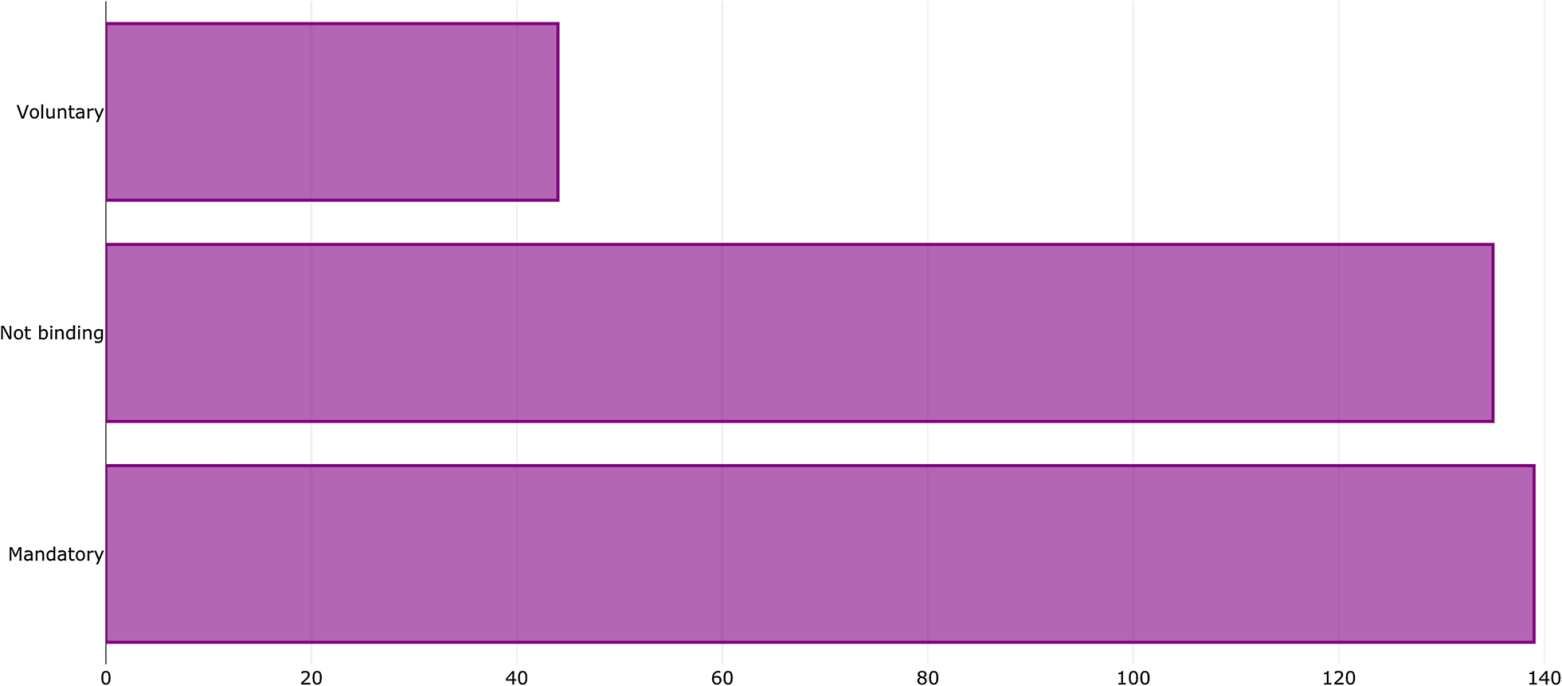
Distribution of policy adoption by authority responsible for the policy’s formulation or promotion. Source: author elaboration

Regarding the policy bindingness, 43% of the measures in the database are mandatory, 42% are non-binding (or no information was obtained about the bindingness), and 14% are voluntary measures (Fig. 5 [Data file 7]). Governments and central banks represent the largest shares of the authorities responsible for the policy's promotion and/or implementation, followed by financial supervisors and regulators (Fig. 6 [Data file 8]).

## Data construction

**Fig. 7:**
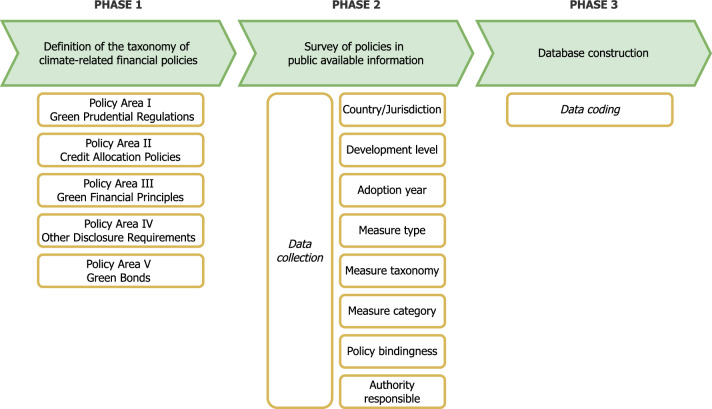
The conceptual framework for developing the global database for climate-related financial policies. Source: author elaboration

The methodology comprises three steps (Fig. 7 [Data file 9]).

**Fig. 8:**
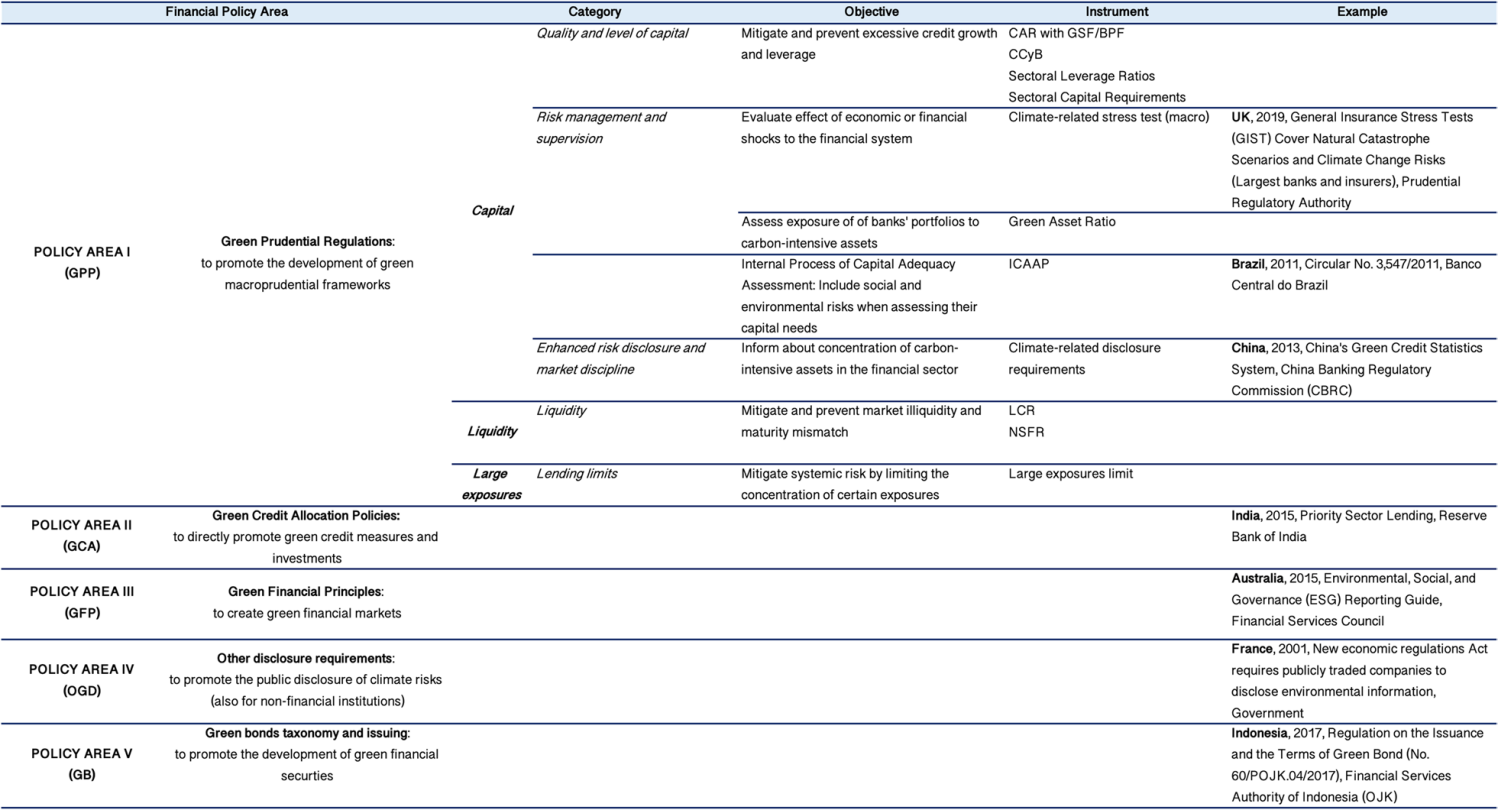
Definition of the five Policy Areas, instruments and objectives of the climate-related policy measures. Source: author elaboration

First, a CRFPs taxonomy is developed (Fig. 8 [Data file 10]). Accordingly, the database covers five policy areas (PA):

- I—Green Prudential Regulations;
- II—Green Credit Allocation Policies;
- III—Green Financial Guidelines;
- IV—Other Green Disclosure Requirements;
- V—Green Bonds Taxonomy and Issuing.

Second, the data collection was designed to gather information on the country/jurisdiction, the economic development level (as defined by the World Bank Indicators), the year of policy adoption, the measure adopted and its bindingness, and the authority responsible for its implementation.

Third, after the information collection, documents relevant to climate-related regulation were sorted by carefully reading the policy text, thus assuring the accuracy of the data collection. This procedure was carefully designed and executed to guarantee thorough coverage and accuracy in the data gathering; the methodology is described in Data file 11. The data has been sample-checked manually when integrated into the database, and each entry is manually validated so, e.g., there are no duplicated counts for each country on a given date.

Table 1 provides an overview of the project and the files available. The overall project is stored and described (Data file 2) in a GitHub repository [8] to allow for regular updates and contributions. The CSV file (Data set 1) is in UTF-8 and comma separated; this allows to import of data into any software. The database is published under the CC BY license.

**Table 1:** Overview of data files/data sets

Label	Name of data file/data set	File types (file extension)	Data repository and identifier (DOI or accession number)
Data set 1	A global database for climate-related financial policies	CSV file	Zenodo https://doi.org/10.5281/zenodo.7567776 [8]
Data file 2	README.dm	.dm	Zenodo https://doi.org/10.5281/zenodo.7567776 [8]
Data file 3	Figure 1.png	.png	Zenodo https://doi.org/10.5281/zenodo.7567776 [8]
Data file 4	Figure 2.png	.png	Zenodo https://doi.org/10.5281/zenodo.7567776 [8]
Data file 5	Figure 3.png	.png	Zenodo https://doi.org/10.5281/zenodo.7567776 [8]
Data file 6	Figure 4.png	.png	Zenodo https://doi.org/10.5281/zenodo.7567776 [8]
Data file 6	Figure 5.pdf	.pdf	Zenodo https://doi.org/10.5281/zenodo.7567776 [8]
Data file 7	Figure 6.png	.png	Zenodo https://doi.org/10.5281/zenodo.7567776 [8]
Data file 8	Figure 7.png	.png	Zenodo https://doi.org/10.5281/zenodo.7567776 [8]
Data file 9	Figure 8.png	.png	Zenodo https://doi.org/10.5281/zenodo.7567776 [8]
Data file 10	Methodology.pdf	.pdf	Zenodo https://doi.org/10.5281/zenodo.7567776 [8]

## Limitations

Existing data do not allow for assessing the effectiveness of climate-related financial policies. D’Orazio and Dirks [5], have made the first contribution in this direction by examining whether the implementation of climate-related financial policies resulted in CO2 emissions reductions in G20 countries. However, more data and information, particularly for some nations, would be required to conduct a more thorough study of the amount of financial resources mobilized by the policies included in the index. Further research in this field may be carried out in the future when more data become available.

## Data Availability

The data described in this Data note can be freely and openly accessed on GitHub under CRFPdata [8]. See Table 1 for details and links to the data.

## Abbreviation

CRFPs: Climate-related financial policies
